# Tandem repeat markers as novel diagnostic tools for high resolution fingerprinting of *Wolbachia*

**DOI:** 10.1186/1471-2180-12-S1-S12

**Published:** 2012-01-18

**Authors:** Markus Riegler, Iñaki Iturbe-Ormaetxe, Megan Woolfit, Wolfgang J Miller, Scott L O’Neill

**Affiliations:** 1 Hawkesbury Institute for the Environment, University of Western Sydney, Locked Bag 1797, Penrith NSW 2751 Australia; 2 School of Biological Sciences, The University of Queensland, QLD 4072, Australia; 3 Center of Anatomy and Cell Biology, Medical University of Vienna, Währingerstr. 10, 1090 Vienna, Austria; 4 School of Biological Sciences, Monash University, Clayton, VIC 3800, Australia

## Abstract

**Background:**

Strains of the endosymbiotic bacterium *Wolbachia pipientis* are extremely diverse both genotypically and in terms of their induced phenotypes in invertebrate hosts. Despite extensive molecular characterisation of *Wolbachia* diversity, little is known about the actual genomic diversity within or between closely related strains that group tightly on the basis of existing gene marker systems, including Multiple Locus Sequence Typing (MLST). There is an urgent need for higher resolution fingerprinting markers of *Wolbachia* for studies of population genetics, horizontal transmission and experimental evolution.

**Results:**

The genome of the *w*Mel *Wolbachia* strain that infects *Drosophila melanogaster* contains inter- and intragenic tandem repeats that may evolve through expansion or contraction. We identified hypervariable regions in *w*Mel, including intergenic Variable Number Tandem Repeats (VNTRs), and genes encoding ankyrin (ANK) repeat domains. We amplified these markers from 14 related *Wolbachia* strains belonging to supergroup A and were successful in differentiating size polymorphic alleles. Because of their tandemly repeated structure and length polymorphism, the markers can be used in a PCR-diagnostic multilocus typing approach, analogous to the Multiple Locus VNTR Analysis (MLVA) established for many other bacteria and organisms. The isolated markers are highly specific for supergroup A and not informative for other supergroups. However, *in silico* analysis of completed genomes from other supergroups revealed the presence of tandem repeats that are variable and could therefore be useful for typing target strains.

**Conclusions:**

*Wolbachia* genomes contain inter- and intragenic tandem repeats that evolve through expansion or contraction. A selection of polymorphic tandem repeats is a novel and useful PCR diagnostic extension to the existing MLST typing system of *Wolbachia*, as it allows rapid and inexpensive high-throughput fingerprinting of closely related strains for which polymorphic markers were previously lacking.

## Background

*Wolbachia pipientis* (α-Proteobacteria) is an obligate endosymbionts of invertebrates, known to infect up to 70% of insect species, as well as spiders, terrestrial crustaceans and medically important filarial nematodes [1-5]. Many strains of *Wolbachia* found in insects manipulate their hosts by inducing feminisation, parthenogenesis, male killing or cytoplasmic incompatibility (CI) [6-9]; in contrast, the *Wolbachia* of nematodes are mutualists necessary for host reproduction [10]. Despite this great diversity of hosts and extended phenotypes, all strains of *Wolbachia* are currently recognised as the single species *W. pipientis.* Within this species, strains are clustered into at least eight divergent clades or 'supergroups', named A to K [11-15].

Several genes have been used for strain typing in *Wolbachia*. Initially, work focused on 16S rDNA[16], the genes encoding the cell division protein, *ftsZ *[11] and the *Wolbachia* surface protein, *wsp *[12]. Subsequent to the demonstration of widespread intra- and intergenic recombination betweens strains [17-19], two multi-locus sequence typing (MLST) systems were developed using different sets of a total of 14 *Wolbachia* genes [20,21]. The MLST approach uses partial nucleotide sequences of several ubiquitous loci with moderate rates of evolution to generate an allelic profile for tested strains. These profiles can be used to type novel isolates, while the relationships between strains may be inferred on the basis of either the allelic profiles themselves or the nucleotide sequences underlying them. MLST data have been used for both strain typing and evolutionary analyses of horizontal transfer events between host species of *Wolbachia* (e.g. [22,23]). Since most MLST primer sets cover housekeeping genes that are under purifying selection, these markers often cannot differentiate between closely related strains. Such difficulties have been revealed in the comparisons between *w*Mel, *w*MelCS and *w*MelPop [20] or *w*Mel and *w*Au within the ST-13 complex which appear indistinguishable in MLST loci [21,24]. These strains induce different phenotypes in their hosts, i.e. *w*Mel induces CI in *Drosophila*, but *w*Au does not [25] and *w*MelPop induces lifespan reduction in its hosts but not *w*Mel [26-28]. The divergence between MLST typing and actual genomic diversity within ST-13 was also raised when these closely related strains were compared for presence or absence of *Wolbachia* prophage WO-A and WO-B [24] and other genomic differences such as a large chromosomal inversion and differential IS5 insertion sites between *w*Mel, *w*MelPop and *w*MelCS [29,30]. Furthermore, MLST can be time consuming and expensive for large population genetic studies as it requires sequencing of all MLST loci for many individuals. Recently other typing systems have been developed for bacteria that build on markers that contain Variable Number Tandem Repeats (VNTR). VNTRs consist of units of DNA (periods) that are tandemly repeated and vary in copy number between different isolates. These loci can be used for a PCR-based typing system and are increasingly being utilised in bacterial strain typing such as Multi Locus VNTR Analysis (MLVA) (e.g. [31-35]). MLVA offers a number of advantages, including highly polymorphic markers that allow fine-scale typing of very closely related isolates, rapid, high-throughput screening that is not dependent on sequencing, and potentially the fingerprinting of multiply infected hosts. The modular structure and evolution of these sites through tandem expansion and contraction also allows cladistic and phylogenetic inference.

Amplicon size polymorphic markers have previously been identified in *Wolbachia* genomes and include transposable element insertion sites [30,36,37], VNTRs [30,38-40] and genes encoding ankyrin repeat domains [36], but their efficiency for strain typing has not yet been compared. In this paper, we used some of these markers in order to estimate the feasibility of a MLVA system for *Wolbachia.* We isolated markers with tandem repeats from the *w*Mel genome [41] and applied them to a number of *Wolbachia* strains from supergroups A, B and C to assess their applicability and resolution for *Wolbachia* strain typing. We chose two types of loci containing tandem repeats, two intergenic VNTR loci and two genes encoding proteins containing ankyrin repeats. The two VNTR loci, VNTR-105 and VNTR-141 were originally isolated from supergroup A strain *w*Mel and were polymorphic between *w*Mel, *w*MelCS and *w*MelPop isolates from different *D. melanogaster* lines [30]. VNTRs are also polymorphic between the closely related *w*Au from *D. simulans* and *w*Wil from *Drosophila willistoni *[38], and serve as highly diagnostic marker sets for fingerprinting conspecific *Wolbachia* strains in the *Drosophila paulistorum* species cluster [39]. Recently, a polymorphic VNTR locus was isolated from supergroup B strain *w*Pip [40]. Ankyrin repeat genes are abundant in the genomes of *Wolbachia* and a number of other intracellular bacteria [42,43]. The number and distribution of these repeats varies substantially between strains that induce different host phenotypes, suggesting that they may be involved in host manipulation [36]. We extended our analysis to include a wider range of *Wolbachia* strains from supergroup A, B and C in order to evaluate the usefulness of the four markers VNTR-105, VNTR-141, *WD0550* and *WD0766*, originally isolated from *w*Mel, in discriminating between *Wolbachia* strains.

## Methods

### *Wolbachia* strains and hosts

We used 14 supergroup A *Wolbachia* isolates from 8 different *Drosophila* species and 2 tephritid species, *Rhagoletis cerasi*, a host that is naturally infected, and *Ceratitis capitata*, microinjected with *Wolbachia* originating from *R. cerasi* (Table 1). Based on previous strain typing using 16S rRNA, ftsZ, wsp and some MLST loci, these 14 strains are moderately or closely related, yet they reveal different phenotypic characteristics, such as varying levels of CI induction (strong, weak, or non-CI inducers), and different CI rescue phenotypes (reviewed in [44]). *Wolbachia* DNA was isolated from *Drosophila* fly stocks reared on standard corn-flour-sugar-yeast medium at 25°C. *Wolbachia*-free controls *D. melanogaster yw*^67c23^T and *D. simulans* Riverside-DSRT were established by tetracycline treatment using standard techniques [45]. *Wolbachia* of *R. cerasi* was isolated from field collected samples from Austria and Hungary [46]. *Wolbachia* from *C. capitata* was isolated from the WolMed 88.6 lab line that was artificially infected with *w*Cer2 from *R. cerasi *[47]. We also included strains from B (*w*No, *w*Bol1, *w*Mau) and C (*w*Dim) supergroups. *w*No and *w*Mau were isolated from *D. simulans*, *w*Bol1 from *Hypolimnas bolina *[48] and *w*Dim from dog heart worm *Dirofilaria immitis *[49].

**Table 1:** List of *Wolbachia* strains.

Strain	Supergroup	Host	Location	mod	res	Reference
***w*Mel**	**A**	*D. melanogaster*	USA	yes	yes	[75,76]
***w*MelCS**	**A**	*D. melanogaster*	CantonS, USA	yes	yes	[30,70]
***w*MelPop**	**A**	*D. melanogaster*	laboratory strain, USA	yes	yes	[26,27]
***w*Au**	**A**	*D. simulans*	Coffs Harbour, Australia	no	no	[25]
***w*San**	**A**	*D. santomea*	Sao Tome, Africa	no*	yes	[77]
***w*Yak**	**A**	*D. yakuba*	Bom Successo, Africa	no*	yes	[77]
***w*Tei**	**A**	*D. teissieri*	Bom Successo, Africa	no*	yes	[77]
***w*Wil**	**A**	*D. willistoni*	Central and South America	no	n.d.	[38]
***w*Spt**	**A**	*D. septentriosaltans*	Central and South America	n.d.	n.d.	[38]
***w*Pro**	**A**	*D. prosaltans*	Central and South America	n.d.	n.d.	[38]
***w*Cer1**	**A**	*R. cerasi*	Hungary	n.d.	n.d.	[46,61]
***w*Cer2**	**A**	*R. cerasi*	Austria	yes	yes	[46,61]
***w*Cer2**	**A**	*D. simulans*	microinjected	yes	yes	[62]
***w*Cer2**	**A**	*C. capitata*	microinjected	yes	yes	[47]
***w*Ri**	**A**	*D. simulans*	Riverside, USA	yes	yes	[16,45]
***w*Ha**	**A**	*D. simulans*	Hawaii, USA	yes	yes	[16,78]
***w*No**	**B**	*D. simulans*	Noumea	yes	yes	[79]
***w*Mau**	**B**	*D. simulans*	microinjected	no	yes	[80]
***w*Bol1**	**B**	*H. bolina*	French Polynesia	yes^¶^	yes^¶^	[81]
***w*Dim**	**C**	*Dirofilaria immitis*	Queensland	no	no	[49]

Modification/rescue phenotypes are included except for strains for which crossing phenotypes had not been determined (n.d.). Modification corresponds to the capacity of a strain to induce cytoplasmic incompatibility (CI) through sperm modification whereas rescue corresponds to the capacity to rescue CI in eggs fertilized by modified sperm [74]. The reference relates to the first description of the strain and/or the phenotype.

* *w*San, *w*Yak, *w*Tei do not induce CI in their original hosts, yet can rescue CI induced by other strains [77], and induce CI in novel hosts upon artificial horizontal transfer through microinjection into *D. simulans *[*23*].

^¶^ CI only expressed in host genotypes that are resistant to the expression of male killing induced by *w*Bol1 [48,81]

### DNA extraction, PCR amplification and sequencing of molecular markers

Total genomic DNA was extracted from either freshly collected specimens or specimens stored in pure ethanol in a -20°C freezer. Extraction was carried out on pools of *Drosophila* flies and single individuals of *Rhagoletis*, *Ceratitis*, *Hypolimnas* and *Dirofilaria*. Flies were homogenized and extracted following either the Holmes-Bonner protocol [50] or the STE extraction method [16]. *Wolbachia* markers were amplified from total genomic DNA using specific primers (Table 2). The *wsp* gene was used as a quality control for DNA extraction and was amplified using the primers 81F and 691R, described in [12]. PCR cycling conditions were as follows: 94°C 3 min, (94°C 30 s, 50°C 30 s, 72°C 3 min) x 35 cycles, then 72°C 10 min. The reaction mixture contained 500 nM of each primer, 200 µM dNTPs, 1.5 mM MgCl_2_, 100 ng of DNA and 1 unit of Taq Polymerase (Promega) in a final volume of 20 µl. The reaction buffer contained 10 mM Tris pH 9.0, 50 mM KCl and 0.1% Triton X-100. PCR products were separated in 1% agarose gels, stained with ethidium bromide and gel-purified using gel extraction kits (QIAGEN). Purified DNA was cloned into the pGEM®-T-easy plasmid (Promega) and sequenced by Macrogen, in Korea, using T7, M13R, and internal primers, as required. Three independent PCRs were sequenced for each gene, checked and confirmed for consistency. Partial sequences of the VNTR-105, VNTR-141 and the ANK genes *WD0550* and *WD0766* from different *Wolbachia* strains have been deposited GenBank database (Table 3).

**Table 2:** List of primers designed according to the *w*Mel genome sequence to amplify VNTRs and ANK genes.

Locus/primer	5’ sequence	Reference
VNTR-141 for	ggagtattattgatatgcg	[30]
VNTR-141 rev	gactaaaggttagttgcat	[30]
VNTR-105 for	gcaattgaaaatgtggtgcc	[30]
VNTR-105 rev	atgacaccttacttaaccgtc	[30]
RO550F	ggccaccatgggatcagaatttgaag	[82]
RO550R	gatgacttatacgcagccccatag	[82]
RO766F	gaccaccatgaaatatgacaaattt	[82]
RO766R	tcaagtaagtgctttttctgtc	[82]

**Table 3:** GenBank accession numbers for VNTR and ANK sequences.

Strain	VNTR-105	VNTR-141	*WD0766*
*w*Mel	JF797619	JF797613	NC_002978*
*w*MelCS	JF797618	JF797611	JF683428
*w*MelPop	as *w*MelCS	JF797612	JF683429
*w*Ri	n.d.	n.d.	NC_012416**
*w*Au	JF797617	JF797608	AY649753
*w*San	JN191623	JN191622	JF683435
*w*Wil	JF797616	JF797607	JF683433
*w*Spt	JF797620	JF797609	JF683431
*w*Pro	n.d.	JF797610	JF683430
*w*Cer1	JF797615	JF797606	JF683434
*w*Cer2	n.d.	JF797614	JF683432
*w*Ha	n.d.	n.d.	JF683436

*wMel genome sequence

**wRi genome sequence

n.d. not determined

### Selection of size variable markers

Polymorphic loci were previously identified from the sequenced genome of *w*Mel of *D. melanogaster* ([41], GenBank reference sequence NC_002978) *in silico* by using Tandem Repeats Finder TRF (http://tandem.bu.edu/trf/trf.html) [51]. Two VNTR regions of interest, VNTR-105 and VNTR-141 were found to be polymorphic between different lines of *D. melanogaster *[30]. The TRF analysis also detected more candidate loci, including some genes encoding ANK domain repeats that can also contain tandemly repeated DNA, and are hence candidate markers for MLVA. Genes encoding ANK domain repeats were previously annotated [41] and variability was found in supergroup A and B *Wolbachia* strains [36]. All of the tandem repeats analysed here were amplified by using primers designed for the conserved flanking regions (single copy coding genes) of the repeats within *w*Mel. We further extended the TRF analysis to other completed *Wolbachia* genomes, *w*Ri ([52] NC_012416), *w*Pip ([53] NC_010981) and *w*Bm ([54] NC_006833) in order to highlight the potential of MLVA for more distantly related *Wolbachia* strains *in silico*. The TRF analysis also included the genomes of *Anaplasma marginale* strain St. Maries (CP_000030) and *Ehrlichia ruminantium* strain Welgevonden (NC_005295) and *Neorickettsia risticii* strain Illinois (NC_013009), the closest relatives of the genus *Wolbachia *[55], as well as a comparison with free living *Escherichia coli* K12 substrain MG1655 (NC_000913). The bacterial genomes were analysed in the basic mode of TRF (version 4.04), with alignment parameters for match, mismatch and indels set at 2, 7 and 7, respectively. The minimum alignment score to report repeats was set at 50, with a maximum period size of 500bp (Table 4).

**Table 4:** Summary of Tandem Repeats Finder (TRF) analysis.

Strain	genome size	TR	TR size in total (% genome)	mean TR period size (range)	mean number of repeats/TR (range)	mean TR internal match (%)
***w*Mel**	1,267,812bp	93	20,349bp (1.6%)	80.9bp (10-291)	2.7 (1.8-11.8)	88.3
***w*Ri**	1,445,904bp	94	16,667bp (1.1%)	58.5bp (10-378)	2.8 (1.8-8.8)	87.5
***w*Pip**	1,482,530bp	72	13,268bp (0.9%)	68.5bp (12-399)	2.8 (1.8-10.6)	87.9
***w*Bm**	1,080,114bp	11	1,032bp (0.1%)	42.8bp (3-112)	3.3 (1.9-15.7)	89.0
*A. m.*	1,197,687bp	54	8,541bp (0.7%)	64.4bp (11-495)	2.8 (1.9-11.2)	91.1
*E. r.*	1,516,355bp	201	95,290bp (6.3%)	138.7bp (1-471)	4.8 (1.8-65.1)	91.6
*N. r.*	879,977bp	27	5,569bp (0.6%)	68.8bp (9-297)	2.9 (1.9-4.9)	88.4
*E. coli*	4,649,675bp	89	17,807bp (0.38%)	70.4bp (8-304)	3.1 (1.9-12.5)	90.1

Analysis in basic TRF basic mode included four completed *Wolbachia* genomes with strain names in bold, *w*Mel (NCBI accession NC_002978), *w*Ri (NC_012416), *w*Pip (NC_010981) and *w*Bm (NC_006833), and the genomes of *Anaplasma marginale* (*A.m.*) strain St. Maries (CP_000030), *Ehrlichia ruminantium* (*E.r.*) st. Welgevonden (NC_005295), *Neorickettsia risticii* (*N.r.*) st. Illinois (NC_013009) and *Escherichia coli* (*E. coli*) K12 substrain MG1655 (NC_000913). TRF detected several tandem repeats (TR) within the same genomic regions, as some tandem repeats contain internal repeats; the number of tandem repeats in column three does hence overrepresent the number of tandem repeat loci in the genome.

### Sequence analysis

The analysis and assembly of the sequences was done using the *EditSeq*, *SeqMan* and *MegAlign* components of the Lasergene sequence analysis software package (DNAStar Inc., Madison, Wis.). The sequenced VNTR loci of the *Wolbachia* strains had to be manually aligned because of their long period length, internal repeats, SNPs and indels within individual VNTR periods. VNTR periods were searched for internal direct repeats, palindromic (dyad) repeats and secondary structures by using DNA Strider [56]. For ANK proteins, domain architecture was predicted using SMART v3.5 (Simple Modular Architecture Research Tool) (http://smart.embl-heidelberg.de/) [57,58] and TMHMM2 (http://www.cbs.dtu.dk/services/TMHMM/). We analysed the phylogenetic relationships between individual ANK repeats from *WD0766* and their orthologs to investigate the mode of evolution of these repeats. All ANK repeats were extracted from the full length sequences of each gene and translated into amino acids. Gaps were inserted where necessary to correct for frameshifts. Sequences were aligned using T_coffee [59]. Maximum likelihood phylogenetic analysis of this alignment was performed using PhyML [60], with a JTT model of amino acid substitution, and a gamma model of rate heterogeneity with four rate classes and the gamma parameter estimated from the data. 1000 bootstrap replicates were performed.

## Results and discussion

### VNTR variability between strains of A-group *Wolbachia*

**Figure 1:**
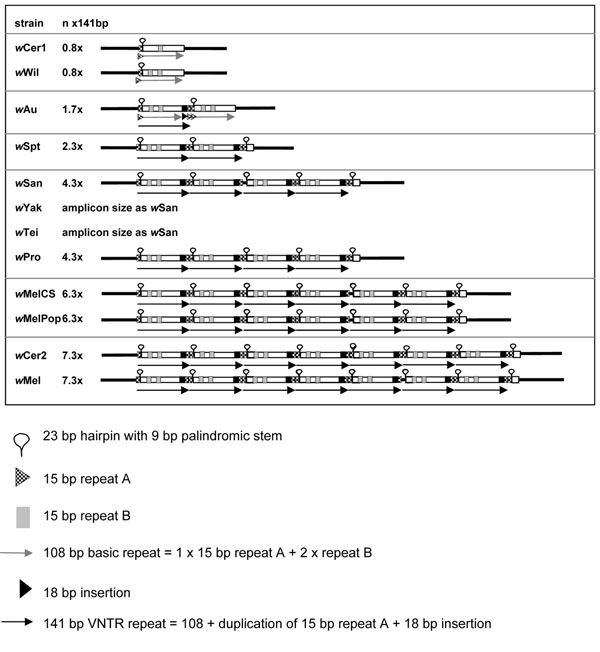
**Schematic presentation of the VNTR-141 locus in ten *w*Mel-like *Wolbachia* strains of *Drosophila* and *R. cerasi*.** The complete 141bp period and the core 108bp period are shown as black and grey arrows, respectively; the 23bp hairpin as a lariat; the two 15bp inverted repeats A and B as dotted and grey boxes, respectively; and the 18bp insertion as a black arrow head.

**Figure 2:**
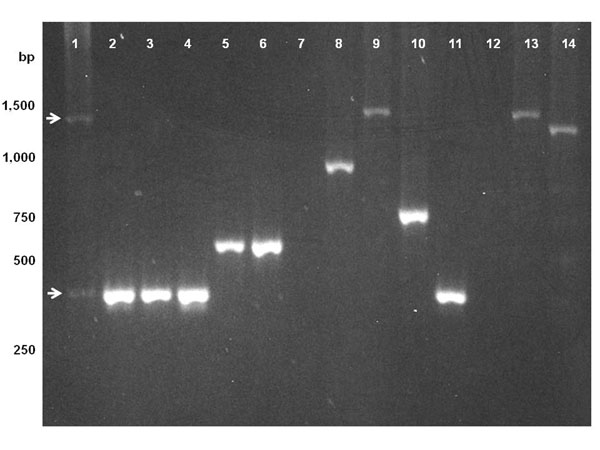
**Diagnostic size difference for the VNTR-141 locus of *Wolbachia*.** Lane **1: ***w*Cer1 and *w*Cer2 doubly infected *R. cerasi* from Austria (the two arrows indicate the two faint bands for *w*Cer1 and *w*Cer2); **2-4**: *w*Wil infected *D. willistoni* from populations collected recently in Panama (Pan98), Mexico (Apa), and Equador (JS); lane **5-6**: *w*Au infected *D. simulans* strain Coffs Harbor and Yaunde 6; lane **7**: uninfected (tetracycline treated) controls = *D. melanogaster* yw^67c23^T; lane **8**: *w*Tei infected *D. teissieri* GN53; lane **9**: *w*Mel infected *D. melanogaster* yw^67c23^; lane **10**: *w*Spt infected *D. septentriosaltans*; lane **11**: *w*Cer1 singly infected *R. cerasi* from Hungary; lane **12**: uninfected (tetracycline treated) control = *D. melanogaster* line yw^67c23^T; lane **13**: *w*Mel infected *D. melanogaster* yw^67c23^; lane **14**: *w*MelCS infected *D. melanogaster* Canton S.

We isolated sequences for two *Wolbachia* VNTR loci, VNTR-141 and VNTR-105, with tandemly repeated periods of 141 and 105bp, respectively, for representative supergroup A *Wolbachia* strains. The loci had previously produced size polymorphic PCR fragments in isolates of *w*Mel and *w*MelCS/*w*MelPop when amplified using primers that were designed to the flanking regions of the two VNTR loci of the sequenced *w*Mel genome [30]. VNTR-141 is positioned between *WD0096* and *WD0098*, and VNTR-105 is between *WD1129* and *WD1131* of the final *w*Mel genome annotation (NCBI accession NC_002978, [41]). The basic 141bp period of VNTR-141 consists of the internal 15bp direct repeat A, a 23bp hairpin with a 9bp palindromic stem, an 18bp insertion and the internal 15bp direct repeat B (Figure 1 of this paper, and Figure 2E of [38]). Diagnostic VNTR-141 PCRs were run on DNA obtained from different *Wolbachia* hosts known to harbour very closely related strains of the symbiont that were not clearly distinguishable by using MLST [20,21,24]. The VNTR-141 fragments were sequenced and compared to the 141bp period of *w*Mel. The shortest VNTR-141 alleles were amplified from *w*Wil and *w*Cer1: they contained only one single period consisting of a 108bp core period without the 18bp insertion, and missing the downstream 15bp A repeat. All other supergroup A strains produced VNTR-141 alleles containing different copy numbers of the 141bp period (Figure 1), i.e. 0.8 (*w*Wil, amplicon size using the locus specific primers 387bp, wCer1 388bp), 1.7 (*w*Au 530bp), 2.3 (*w*Spt 643bp), 4.3 (*w*San 889bp, *w*Pro 925bp; *w*Yak and *w*Tei had similar amplicon sizes to *w*San but were not sequenced), 6.3 (*w*MelCS 1189bp, *w*MelPop 1189bp) and 7.3 (*w*Mel 1330bp, *w*Cer2 1348bp for both original host *R. cerasi* and novel host *C. capitata*) (Figure 1). These polymorphic amplicons in VNTR-141 were visualised by standard PCR as different amplicon sizes on an agarose gel (Figure 2). Multiply infected *R. cerasi *[46,61] revealed two bands, with amplicons representing *w*Cer1 and *w*Cer2 (Figure 2). The VNTR alleles of *w*Cer2 were assigned through comparisons with the isolates from the microinjected novel hosts *D. simulans *[62] and *C. capitata *[47]. Besides the internal deletions in the *w*Wil and *w*Cer1 periods, and variation in copy numbers, the sequence composition of the VNTR-141 periods are almost identical (i.e. 99%) within *w*Mel and other strains, and hence highly conserved. For this reason a phylogenetic sequence analysis, other than the analysis of repeat numbers in cladistical approaches, is not informative.

**Figure 3:**
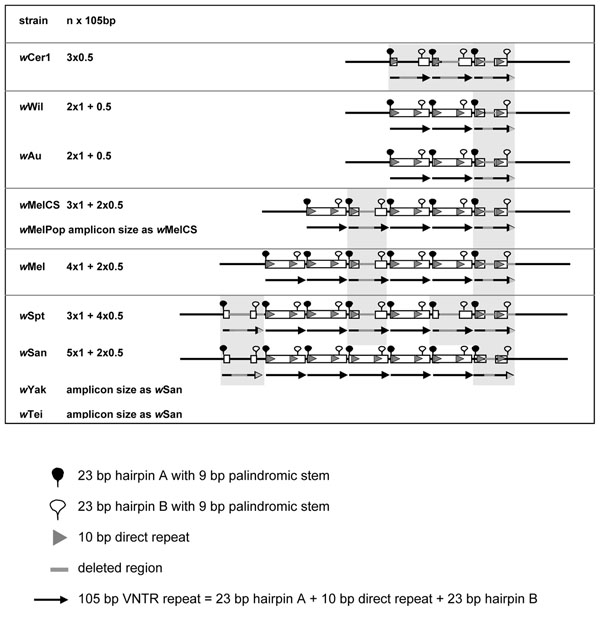
**Schematic presentation of the VNTR-105 locus in seven *w*Mel-like *Wolbachia* strains of *Drosophila*.** The complete 105bp period is shown as black arrows; the two 23bp hairpins A and B as full and empty lariats, respectively; the 15bp inverted repeat as grey boxes; and deleted sections in grey.

In contrast to VNTR-141, the basic period of VNTR-105 is 105bp long containing two 23bp hairpins with 9bp palindromic stem structures and one internal short repeat of 10bp (Figure 3). VNTR-105 of *w*Mel contains four complete 105bp periods, and two with internal deletions of 25bp each. *w*MelCS and *w*MelPop lack one of the complete 105bp periods, i.e. contain three complete 105bp copies and two with internal deletions of 32bp (Figure 3). The tested supergroup A strains display different alleles in the VNTR-105 locus with amplicon sizes ranging from 3x0.5 copies (*w*Cer1, amplicon size using the locus specific primers 998bp), 2.5 copies (*w*Wil 1065bp, *w*Au 1065bp), 3+2x0.5 copies (*w*MelCS and *w*MelPop 1241bp), 4+2x0.5 copies (*w*Mel 1347bp), 3+4x0.5 copies (*w*Spt 1408bp) and 5+2x0.5 copies (*w*San, 1476bp; *w*Yak and *w*Tei had similar amplicon sizes to *w*San but were not sequenced). *w*Cer2 had a large amplicon for this VNTR locus and difficulties were experienced with accurately sequencing these large loci because of restrictions with read lengths, as well as problems in detecting an accurate overlap between forward and reverse sequences. VNTR-105 amplicon size differences can be easily resolved on agarose gels (data not shown). In comparison to VNTR-141, the structure of the VNTR-105 locus is less conserved within and between strains because of internal deletions, yet the period sequences are almost identical (i.e. 98%) within *w*Mel and between other strains. For this reason a phylogenetic analysis of period sequence data is not appropriate, whereas the analysis of diagnostic characters such as copy numbers are more informative (Figure 3).

We extended our PCR analysis to a wider range of *Wolbachia* strains, including *w*Ri and *w*Ha, both supergroup A strains that are distantly related to *w*Mel, as well as strains from supergroup B (*w*No, *w*Bol1, *w*Mau) and C (*w*Dim). None of these strains yielded PCR products for the tested VNTR primers, probably because of sequence divergence within the primer region or genome rearrangements [52-54]. Because of the latter it was not attempted to design primers of conserved coding regions in distantly related strains.

### Evolution of repeats in VNTR loci

The individual periods of VNTR-141 and VNTR-105 respectively display high sequence conservation within and between strains, with variability in the copy numbers and internal deletions within some of the repeated periods. Two evolutionary processes may be shaping these loci with high variability in repeat copy numbers yet small sequence divergence. The accumulation of tandemly repeated periods may be facilitated through slippage and mispairing in the process of *Wolbachia* DNA replication and repair. Slipped-strand mispairing has previously been identified as a source for generation of repeat copies in general [63-65] and in *E. ruminantium* in particular, a genome with an elevated number of tandem repeats [66]. Palindromic sequences with the strong potential of forming secondary stem loops are well known to cause slipped-strand mispairing [67]. Hence we assume that the hairpins present in both *Wolbachia* VNTRs may trigger slippage in both these loci. The second evolutionary mechanism in action could be concerted evolution between different periods within the two loci, a phenomenon that has previously been observed in members of gene families that tend to be more similar within a species than between species because of the elimination or fixation of new point mutations [68]. The high structural turnover, triggering expansions and/or contractions of copy numbers in both VNTR loci of *w*Mel-like *Wolbachia*, can thus be applied for simple and rapid but highly informative symbiont fingerprinting by standard PCR (Figure 2). We cannot infer directionality between expansion and contractions in the evolution of both loci. It is hence impossible to determine whether low copy numbers within the intergenic loci manifest an ancestral or derived state. It has been suggested though that tandem repeats go through cycles of gradual expansion followed by collapse of repeats [69]. It is hence adequate to state that closely related strains are more likely to have similar copy numbers, e.g. *w*Mel and *w*MelCS. Interestingly, the CI inducing strains *w*Cer2, *w*Mel and *w*MelCS contain larger VNTR loci when compared to the non CI inducing *w*Wil and *w*Au, with larger VNTR loci in *w*Mel than *w*MelCS that coincide with stronger CI induction in *w*Mel than *w*MelCS [70]. Furthermore increased copy numbers in one locus correspond with increased copy numbers in the second. Such a coincidence of intergenic tandem repeat variation with CI phenotype was also observed for supergroup B *Wolbachia* in *C. pipiens*[40]. Yet, these observations are not sufficiently supported by replication to conclude about any potential links between genotypes and phenotypes, but they warrant further structural and functional studies of the VNTR repeat expansions.

### ANK gene variability between strains of A-group *Wolbachia*

Unlike most bacteria, genes that encode proteins with ANK repeats are extremely abundant in *Wolbachia*, representing up to 2-4% of the total number of genes in *w*Mel [41], *w*Ri [52] and *w*Pip [53,71]. Some of the variability in these genes appears to correlate with crossing types in mosquitoes [72]. Several of the 23 ANK genes initially annotated in the *w*Mel genome are highly variable between the CI-inducing strain *w*Mel and the non-CI inducing related strain *w*Au [36]. These differences included point mutations, frameshifts and premature stop codons, presence/absence of transmembrane domains, disruption by insertion elements and variability in the number of predicted ANK repeats in the encoded proteins.

Based on earlier work [36], we performed an initial PCR screening (data not shown) using the most variable *w*Mel ANK genes (*WD0035*, *WD0294*, *WD0385*, *WD0498*, *WD0514*, *WD0550*, *WD0636*, *WD0766* and *WD1213*- also see results of TRF analysis below) in order to look for size differences across the *Wolbachia* strains used in this study. Some of the ANK genes could not be amplified in all strains, probably due to sequence divergence. For the ones that could be amplified, the non-phage related ANK genes *WD0550* and in particular *WD0766* were found to be the most variable in terms of size difference among the *Wolbachia* strains and they were selected for further analysis, with sequence data reported for *WD0766* only.

**Figure 4:**
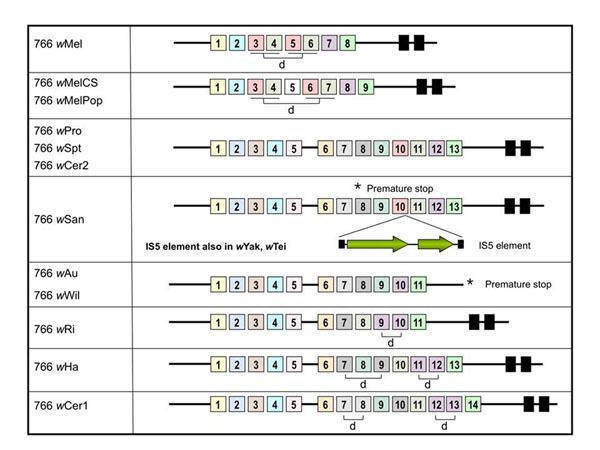
**Domain architecture of the WD0766 ANK domain protein in *Wolbachia* strains.** The location of ANK motifs (coloured boxes with numbers) was determined using SMART v3.5 (http://smart.embl-heidelberg.de/). Transmembrane domains (black boxes) were predicted using the TMHMM2 server. The presence of a frameshift in the *w*Au and *w*Wil WD0766 gene creates a premature stop (*) that prevents the translation of the transmembrane domains. The *w*San, *w*Yak and *w*Tei genes also contain a premature stop (*) that prevents the translation of 6 ANK domains and two transmembrane domains. These genes also contain an IS5 element insertion inside the 10^th^ ANK domain. Some of the ANK repeat motifs are duplicated (d). The colour scheme corresponds to the DNA sequence similarity of the ANK repeat motifs (Figure 5).

In *w*Mel, WD0766 encodes a 51.8kDa protein containing eight ANK repeats and two transmembrane domains (TMDs) in the C-terminus. When this gene was sequenced in several *Wolbachia* strains, the number of predicted ANK repeats was found to be quite different among them, ranging from eight repeats in *w*Mel to 14 in *w*Cer1 (Figure 4). The *w*Au, *w*Wil and *w*Ri strains contained 11 ANK repeats, but the proteins were truncated by a premature stop codon that resulted in the elimination of the predicted TMDs in *w*Au and *w*Wil. *WD0766* in *w*San is disrupted by a premature stop after the seventh ANK domain and contains a 918bp IS5 insertion element in the middle of its 10^th^ ANK repeat (Figure 4). PCR results (data not shown) suggest that this IS5 insertion is also present in the orthologous gene in *w*Yak and *w*Tei, but these amplicons were not sequenced. The sequence of the *w*San IS5 element is identical to that of the 13 IS5 elements present in the *w*Mel genome [41]. Disruption of a *Wolbachia* ANK gene by an IS5 insertion element has previously been observed in the *WD0385* gene from *w*Au (GenBank AY664873) [36], although in this case the insertion sequence differs by 5 nucleotides from the *w*Mel and *w*San IS5 elements. *w*Spt, *w*Cer2 and *w*Ha strains had the same structure for the *WD0766* proteins (13 ANK domains + 2 TMDs), whereas the *w*Cer1 protein contained 14 ANK domains and 2 TMDs.

**Figure 5:**
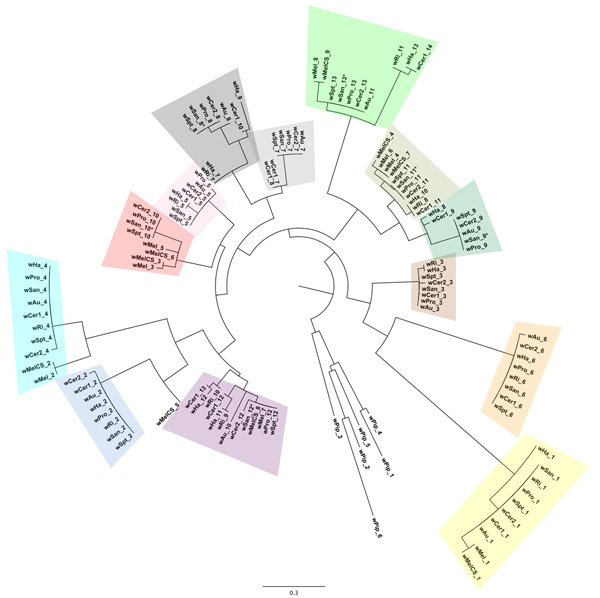
**Maximum likelihood phylogeny of individual ANK repeats from WD0766 and its orthologs.** Names indicate the strain of *Wolbachia* and the repeat number, as labelled in Figure 4. The scale bar corresponds to nucleotide substitutions per site.

*WD0550* was also found to be variable among the strains analysed, although it was not as informative as *WD0766*. For this reason only a subset of strains was analysed for this locus in more detail. *WD0550* codes for a 36.4kDa protein containing six predicted ANK repeats and has no TMDs. The protein contains six ANK repeats in *w*Mel and *w*Spt, and eight repeats in *w*MelCS, *w*San, *w*Cer2, *w*Au and *w*Wil (data not shown).

### Evolution of repeats in *WD0766*

**Figure 6:**
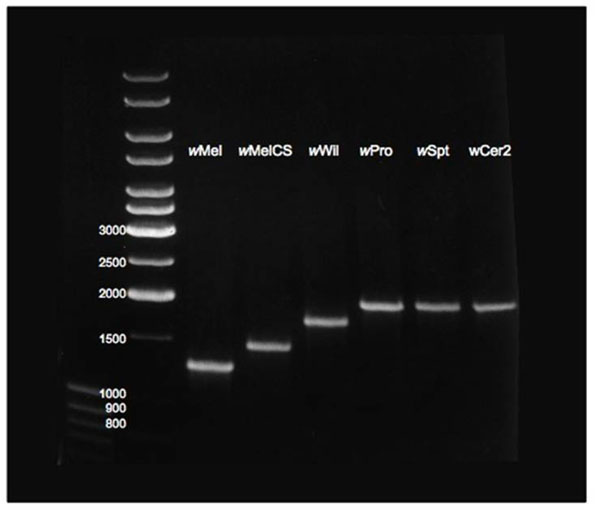
**Diagnostic size polymorphism of the *WD0766* gene.** Isolates include *Wolbachia* of *D. melanogaster* (*w*Mel, *w*MelCS), *D. willistoni* (*w*Wil), *D. prosaltans* (*w*Pro), *D. septentriosaltans* (*w*Spt) and *D. simulans* transinfected with *Wolbachia* from *R. cerasi* (*w*Cer2).

Orthologs of *WD0766* encode for proteins containing different numbers of ANK repeats in different *Wolbachia* strains. Additional repeat copies may be gained by the duplication or loss of single or multiple repeats, and genes containing these repeats may also diverge due to loss or shuffling of repeat periods. To investigate the patterns of change in the number and order of ANK repeats in these proteins, we aligned the amino acid sequences of all individual repeats and performed a maximum likelihood analysis of the phylogenetic relationships between them (Figure 5). The tree shows clusters of typically six to ten repeats, separated by relatively long internal branches. Despite the large ratio of internal to tip branch lengths, bootstrap values on this tree are almost all extremely small, probably due to the short length of the alignment (34 residues). However, a clear pattern is observed wherein repeats in similar positions within multiple orthologs cluster together. For example, the first ANK repeat present in every ortholog clusters in a single clade, marked in yellow in Figures 4 and 5. A similar clustering is seen for the last repeat of every ortholog (marked in green), and for the second repeat in *w*Mel and *w*MelPop/*w*MelCS with the fourth repeat of all other orthologs (marked in blue). Figure 4 shows the structure of each ortholog, with repeats that cluster together in the tree coloured in the same shade. Similar to VNTRs, ANK loci of *Wolbachia* provide highly informative and strain-specific marker sets that allow easy separation via PCR and high-resolution diagnosis of host infections (Figure 6).

A number of inferences about the evolution of the ANK repeats in these genes can be drawn from the tree in Figure 5 and the mapping of the phylogenetic data onto the modular structure of the genes. First, it is likely that the ancestral copy of this gene at the base of supergroup A already contained most of the repeats seen today, probably in a very similar linear order. Most of the clusters in the tree contain repeats from 7 or more of the orthologs, and the order of these orthologous repeats along the genes is highly similar. There is only one clear example of repeat shuffling: the eighth and ninth repeats in the *w*Pro/*w*San/*w*Au groups occur in the reverse order in wCer1 (as repeat periods 10 and 9), while *w*Ha may represent an intermediate stage, with the repeats orthologous to *w*Pro 8 and 9 followed by a second copy of a repeat orthologous to *w*Pro 8. Secondly, at least some variation in repeat number is due to lineage-specific tandem duplication of a single repeat (e.g. repeats 7 and 8 in *w*Cer1) or of multiple repeats (repeats 3-4 and 5-6 in *w*Mel).

### Extension of MLVA markers to other *Wolbachia* supergroups

In comparison to the MLST markers, the highly polymorphic markers used here have a major trade-off in the loss of universal applicability for all *Wolbachia* strains. Here we have focused on *Wolbachia* supergroup A and tested the primers of these markers in other supergroups but primers did not amplify the loci or the loci were not informative. The presence of VNTR loci was restricted to subsets of supergroup A while genes containing ANK domain repeats were found in all supergroup A strains. *In silico* analysis of three other completed genomes, *w*Ri, *w*Pip and *w*Bm of supergroups A, B and D, respectively, revealed though that tandem repeated regions occur throughout these supergroups and may be of relevance for MLVA in other supergroups. As further genome data become available it will be possible to extend this to an even larger group of *Wolbachia* isolates. A TRF analysis of *w*Mel revealed 93 sites with direct tandem repeats of periods ranging from 10bp to 291bp, with internal match percentages from 68% to 100% (Table 4). The larger *w*Ri genome has a similar number of tandem repeats while *w*Pip has a smaller set of tandem repeats. The tandem repeats of *w*Mel, *w*Ri and *w*Pip have similar characteristics such as comparable period sizes, copy numbers as well as internal match ratios (Table 4). The number of tandem repeats in *w*Bm is reduced by a factor of 10 when compared with the supergroup A and B *Wolbachia*, and the tandem periods appear to be shorter. This reduction in *w*Bm is in accordance with the earlier described higher rate of secondary genome reduction in this strain [54]. Within the group of the closest relatives of the genus *Wolbachia*, the sequence of *E. ruminantium* revealed the highest content of tandem repeats for bacteria reported so far (Table 4), with size polymorphism in tandem repeats within the isolate that was used for genome sequencing the genome [66]. Our *in silico* analysis predicted the presence of variable tandem repeat markers in supergroup A strains and could hence readily be developed and tested on *Wolbachia* isolates from other supergroups. Highly polymorphic markers will be useful in population dynamic and population genetic studies similar to the ones undertaken in *w*Mel-like strains [30,38,39]. We have not analysed the unfinished genome data sets of *Wolbachia* (e.g. [73]). A large proportion of tandem repeats are located in intergenic regions that tend to be assembled in genome sequencing projects last, yet their conserved flanking regions are required for the isolation of VNTR markers from total genomic extracts. A polymorphic VNTR locus has recently been reported for a supergroup B strain after applying a similar approach to *w*Pip isolated from different *C. pipiens* populations [40].

Interestingly, our TRF analysis only detected five ANK repeat regions (*WD0294*, *WD0385*, *WD0514*, *WD0550* and *WD0766*) of the 23 annotated genes encoding ANK repeat domains. Coincidentally, this group of genes includes the most variable genes encoding ANK repeat domains, suggesting that repeat extension/contraction is a strong diversifying mechanism in these genes.

Most of the primers designed for *w*Mel ANK genes amplified expected PCR amplicons from supergroup A *Wolbachia*, but not from the majority of supergroup B, probably due to sequence divergence [36]. ANK domain genes are known to be present in other *Wolbachia* groups. In the B group mosquito strain *w*Pip that infects mosquitoes there are 60 genes encoding ANK repeats, some of them also variable [53,71,72], whereas the fully sequenced D group *w*Bm strain that infects the nematode *Brugia malayi* contains 5 ANK genes and 7 related pseudogenes [54]. Although *w*Mel ANK genes were used as a reference in our study, another A group *Wolbachia* strain, *w*Ri, contains 35 ANK genes, some of them very distinct from the *w*Mel genes, probably as a result of duplications and recombination events [52]. Partial sequences of other A group strains have also revealed high numbers of ANK genes [73]. Thus, it seems clear that ANK genes are a signature feature in *Wolbachia* that can be potentially utilised to fingerprint closely related strains in A and other groups.

## Conclusion

The identification of amplicon size polymorphic markers of *Wolbachia* provides a valuable addition to existing typing systems such as MLST, for the following three reasons: (1) The MLVA markers presented here display higher rates of evolution than the MLST loci, which are conserved protein encoding genes. Using MLVA, *Wolbachia* strains clustered in the same groups as in MLST typing, yet with a higher resolution that could be useful for different types of questions that MLST has not yet been able to target. These questions include the study of *Wolbachia* population genetics within infected species [30,38,39], and will further extend studies of horizontal transmission between host species for which MLST was originally developed [22]. Highly polymorphic markers will also be useful for experimental evolution of *Wolbachia* in order to track small genomic changes in short time frames. This higher resolution comes with the cost though, that markers are not universally applicable to the entire diversity of *Wolbachia*. (2) The majority of *Wolbachia* genomes are dotted with many different repeat regions which are highly appropriate to be targeted for the isolation of possible polymorphic markers. Tandem repeat markers such as the ones developed here can be tailored to individual studies. (3) MLVA markers are ideal for rapid and high-throughput DNA fingerprinting, as no sequencing is required. The markers are ideal to detect multiple infections in single PCR reactions if strains contain alleles with variable amplicon sizes. Our analysis of the evolution of the tandem repeat regions shows that they evolve by gain or loss of repeats. The variability in the number of ANK repeats, generally constituted by 33 amino acids each, creates size differences that are multiples of 99bp and, like VNTRs consisting of >100bp periods, can be clearly identified following simple PCR screenings without the need of initial sequencing or RFLP analyses as in the case of point mutations. The use of 2-3 highly variable markers per strain can generate easily readable fingerprints.

## List of abbreviations used

CI: cytoplasmic incompatibility; MLVA: multiple locus variable number tandem repeat analysis; MLST: multiple locus sequence typing; VNTR: variable number tandem repeat; ANK: ankyrin domain; TRF: tandem repeats finder.

## Competing interests

The authors declare that they have no competing interests.

## Authors’ contribution

MR, IIO, WJM and SLO had the initial idea for this manuscript. MR, IIO, WJM and SLO designed the study. MR, IIO and WJM performed laboratory work. MR, IIO, WJM, MW performed data analysis. MR, IIO, WJM, MW and SLO wrote the manuscript. All authors approved the final manuscript.
